# Synthesis and crystal structure of a new isomer of poly[di-μ_3_-cyanido-μ-2,6-di­methyl­pyrazine-dicopper(I)]

**DOI:** 10.1107/S2056989025006267

**Published:** 2025-07-23

**Authors:** Christian Näther

**Affiliations:** a Institut für Anorganische Chemie, Universität Kiel, Max-Eyth.-Str. 2, 24118 Kiel, Germany; Universidad de la República, Uruguay

**Keywords:** synthesis, crystal structure, copper(I) cyanide, 2,6-di­methyl­pyrazine, coordination polymer

## Abstract

The title compound, [Cu_2_(CN)_2_(C_6_H_8_N_2_)]_*n*_ or Cu_2_(CN)_2_(2,6-di­methyl­pyrazine), was prepared by the reaction of copper(I) cyanide with 2,6-di­methyl­pyrazine in water. Its asymmetric unit consists of two crystallographically independent copper(I) cations and cyanide anions as well as one crystallographically independent 2,6-di­methyl­pyrazine ligand in general positions. Each copper cation is fourfold coordinated by one N atom of the 2,6-di­methyl­pyrazine ligand and three cyanide anions, that are disordered so that each C and N position has mixed occupancy with a ratio between N and C of 94:6 and 77:23. The copper cations are linked by the cyanide atoms into layers that are further connected into a 3D network by the 2,6-di­methyl­pyrazine ligands. Powder X-ray diffraction (PXRD) proves that a pure crystalline phase has been obtained. It is noted that this crystal structure represents a new isomer of Cu_2_(CN)_2_(2,6-di­methyl­pyrazine), which has already been reported in the literature [Chesnut *et al.* (2001 ▸). *J. Chem. Soc. Dalton Trans*. pp. 2567–2580].

## Abstract 2

In the crystal structure of the title compound, the copper(I) cations are linked by the cyanide anions into layers that are additionally connected by the 2,6-di­methyl­pyrazine ligands into a 3D network. The title compound represents a new isomer of Cu_2_(CN)_2_(2,6-di­methyl­pyrazine), which has already been reported in the literature.

## 1. Chemical context

Coordination compounds based on copper(I) halides and pseudohalides show a pronounced structural variability and therefore, have been investigated for many decades (Kromp & Sheldrick, 1999 ▸; Peng *et al.*, 2010 ▸; Näther *et al.*, 2002 ▸, 2017 ▸; Li *et al.*, 2005 ▸). Such compounds usually consist of Cu*X* subunits (*X* = Cl, Br, I, CN, SCN) that are linked into mono- or di-periodic coordination networks that can further be expanded when bridging coligands are used. In most cases, such compounds are prepared in solution but we have found that new compounds with more condensed Cu*X* networks can also be prepared by thermal decomposition of suitable precursor compounds (Näther *et al.*, 2001 ▸; Näther & Jess, 2004 ▸).

In the course of our systematic work, we became inter­ested in compounds with 2,6-di­methyl­pyrazine that can act as a bridging ligand, but also as a terminal ligand because the second N atom is sterically shielded by the two neighbouring methyl groups. Compounds with copper(I) halides and 2,6-di­methyl­pyrazine have been already reported. These include Cu_2_Cl_2_(2,6-di­methyl­pyrazine (Refcode YEFPOR; Fan *et al.*, 2015 ▸), as well as CuI(2,6-di­methyl­pyrazine) [Refcodes TONQOE (Kitada & Ishida, 2014 ▸) and TONQOE01 (Zhang *et al.*, 2014 ▸)]. Moreover, one pseudo halide compound with the composition Cu_2_(CN)_2_(2,6-di­methyl­pyrazine) is also reported (Refcode SUYGAU; Chesnut *et al.*, 2001 ▸). In the CuI compound, the ratio between the cation and the anionic ligand is 1:1, whereas in the CuCl and CuCN compounds it is 2:1. Therefore, it can be assumed that for the latter two compounds with chloride and cyanide, a further 2,6-di­methyl­pyrazine-rich phase might exist that can be transformed into the better known 2,6-di­methyl­pyrazine-deficient phases. Such compounds would be of inter­est for their thermal reactivity. In the beginning, we focused on compounds with CuCN, which was reacted in different ratios with 2,6-di­methyl­pyrazine. In one of these batches, we obtained crystals that were characterized by single-crystal X-ray diffraction, which proved that a new isomer of Cu_2_(CN)_2_(2,6-di­methyl­pyrazine) had accidentally formed. Later we have found that this compound can be prepared pure if CuCN and 2,6-di­methyl­pyrazine are reacted in a 2:1 ratio at room-temperature.
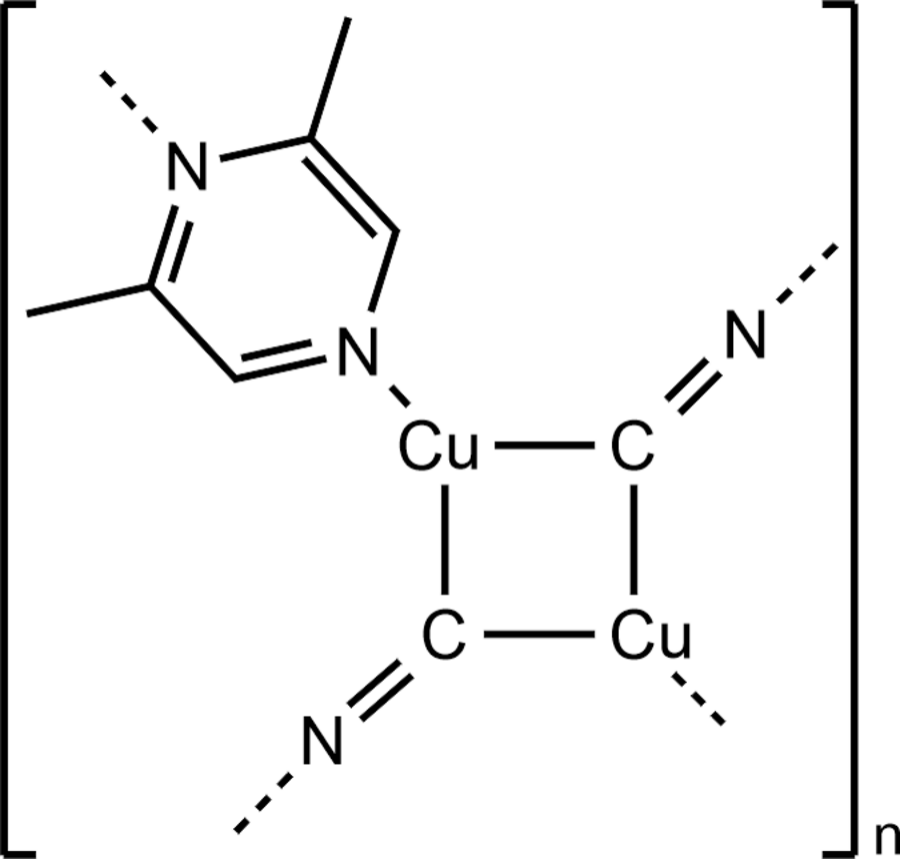


## 2. Structural commentary

**Figure 1:**
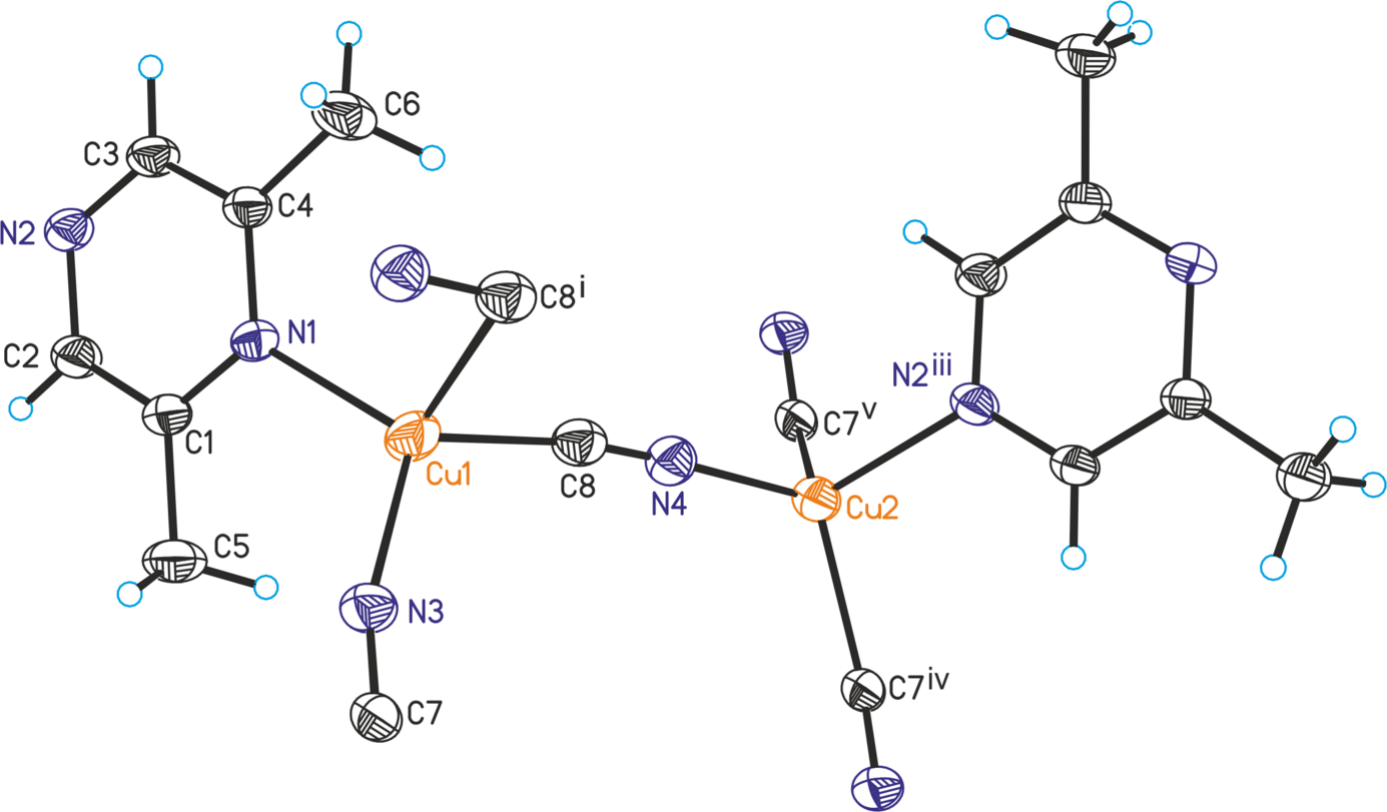
Crystal structure of the title compound with labeling and displacement ellipsoids drawn at the 50% probability level. Symmetry codes for the generation of equivalent atoms: (i) −*x* + 1, −*y* + 1, −*z* + 1; (iii) −*x* + 1, *y* + 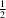, −*z* + 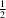; (iv) −*x* + 2, −*y* + 1, −*z* + 1; (v) *x*, *y*, *z* − 1. Please note that the cyanide anions are partly disordered so that the N and C atoms occupy the same crystallographic positions. This disorder is not considered in the labeling of the atoms.

**Figure 2:**
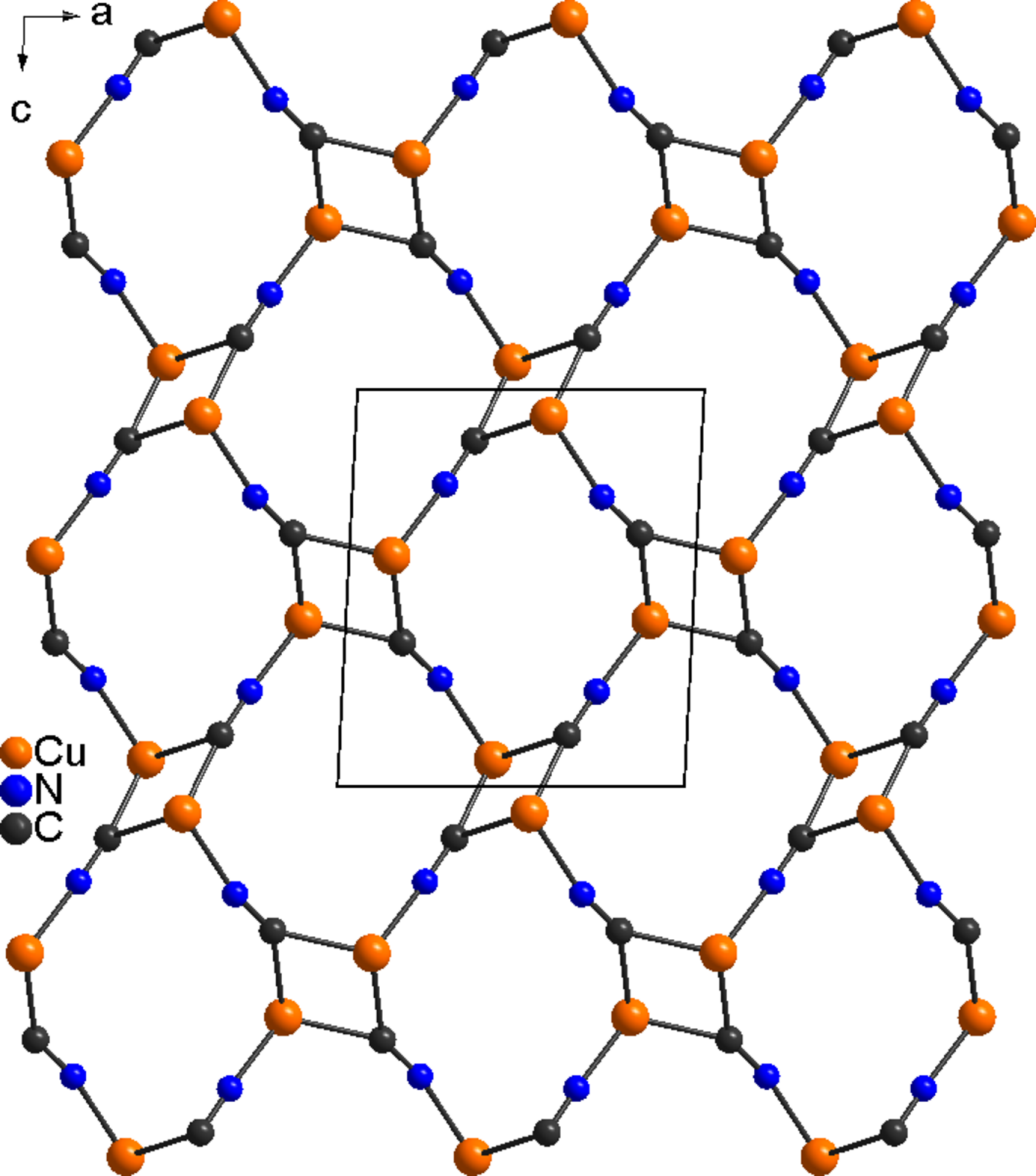
View of the CuCN network in the title compound in a view along the crystallographic *b*-axis direction.

**Figure 3:**
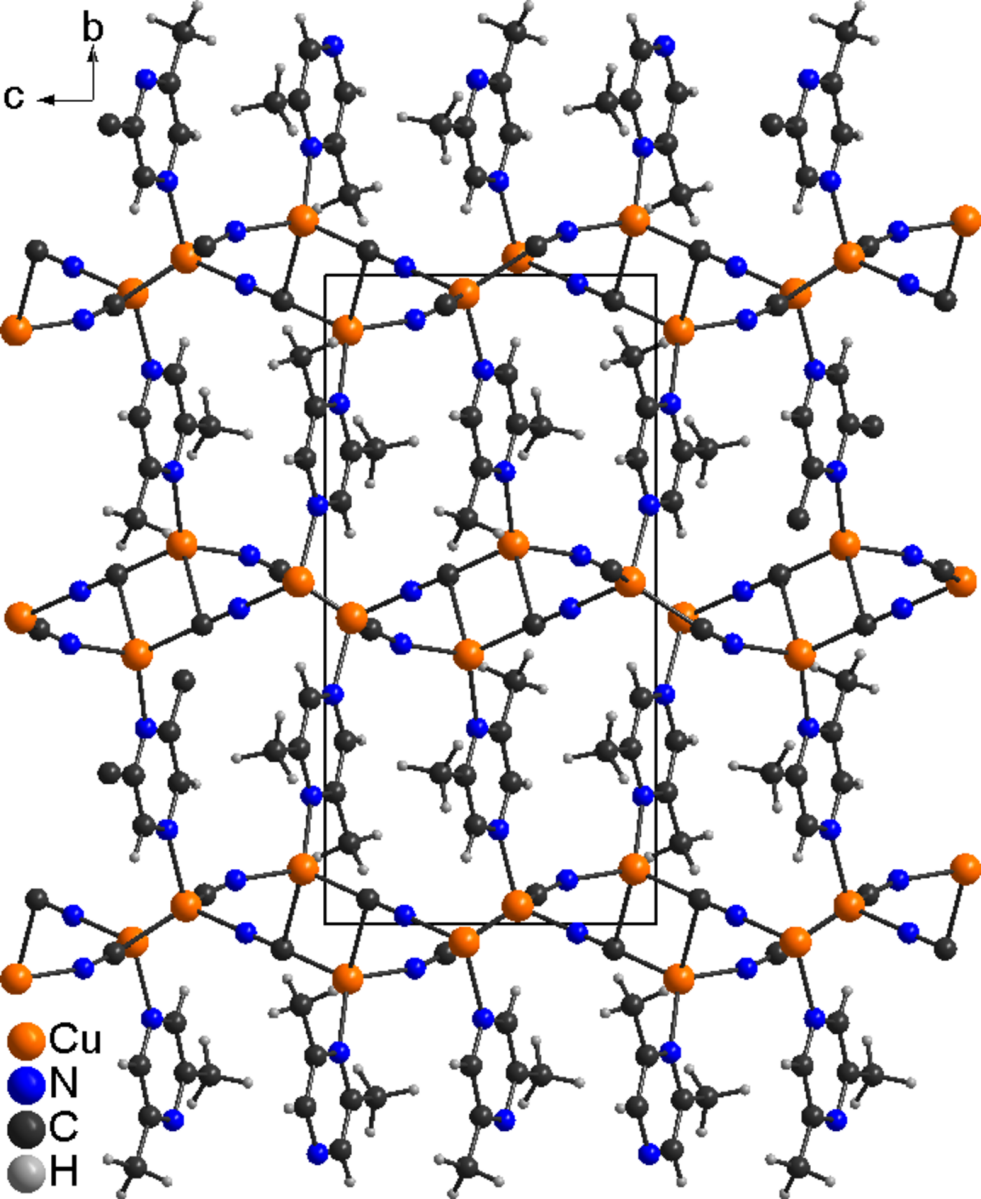
Crystal structure of the title compound in a view along the crystallographic *a*-axis direction.

The asymmetric unit of the title compound, Cu_2_(CN)_2_(2,6-di­methyl­pyrazine), consists of two crystallographically independent copper cations, two independent cyanide anions and one independent 2,6-di­methyl­pyrazine ligands, all of them located in general positions (Fig. 1 ▸). The cyanide anions are partly disordered so that the C and the N atoms occupy the same crystallographic position. Each copper cation is fourfold coordinated by three cyanide anions and one 2,6-di­methyl­pyrazine ligand, but for Cu1 one relatively long Cu—C distance to a symmetry-related cyanide anion is observed, which is at the limit of that expected for a typical coordinative bond (Table 1 ▸). From the bond angles, it is obvious that a distorted tetra­hedral coordination is present, as expected for copper(I) cations (Table 1 ▸). Each of the two copper cations is linked by two cyanide anions into four-membered rings built up of Cu_2_(CN)_2_ units that are linked by the anionic ligands to neighboring Cu_2_(CN)_2_ units (Fig. 2 ▸). The Cu⋯Cu distances within these rings are 3.0003 (7) and 2.4031 (7) Å (Table 1 ▸). Four such units form twelve-membered rings that condense into layers that are parallel to the (010) plane (Fig. 2 ▸). Neighboring layers are connected by bridging 2,6-di­methyl­pyrazine ligands, which are oriented along the crystallographic *b*-axis direction, forming a three-dimensional coordination network (Fig. 3 ▸).

The crystal structure of the title compound is different from that of the isomer of Cu_2_(CN)_2_(2,6-di­methyl­pyrazine) that has already been reported in the literature (Chesnut *et al.*, 2001 ▸). The asymmetric unit of this compound also consists of two crystallographically independent copper cations, but one of them is only threefold coordinated, whereas the second cation is fourfold coordinated. The CuCN network of this compound also consists of Cu_2_(CN)_2_ units that are linked into twelve-membered rings, but these rings do not condense into layers and instead CuCN double chains are formed. Nevertheless, because of the bridging 2,6-di­methyl­pyrazine ligands, a 3D network is also formed. Finally, it is noted that this isomer was prepared under hydro­thermal conditions at 453 K, which indicates that the title compound is thermodynamically stable at least at room-temperature.

## 3. Supra­molecular features

The crystal structure of the title compound is exclusively dominated by coordinative bonds. There are no other directional inter­actions such as, for example, hydrogen bonding.

## 4. Database survey

**Figure 4:**
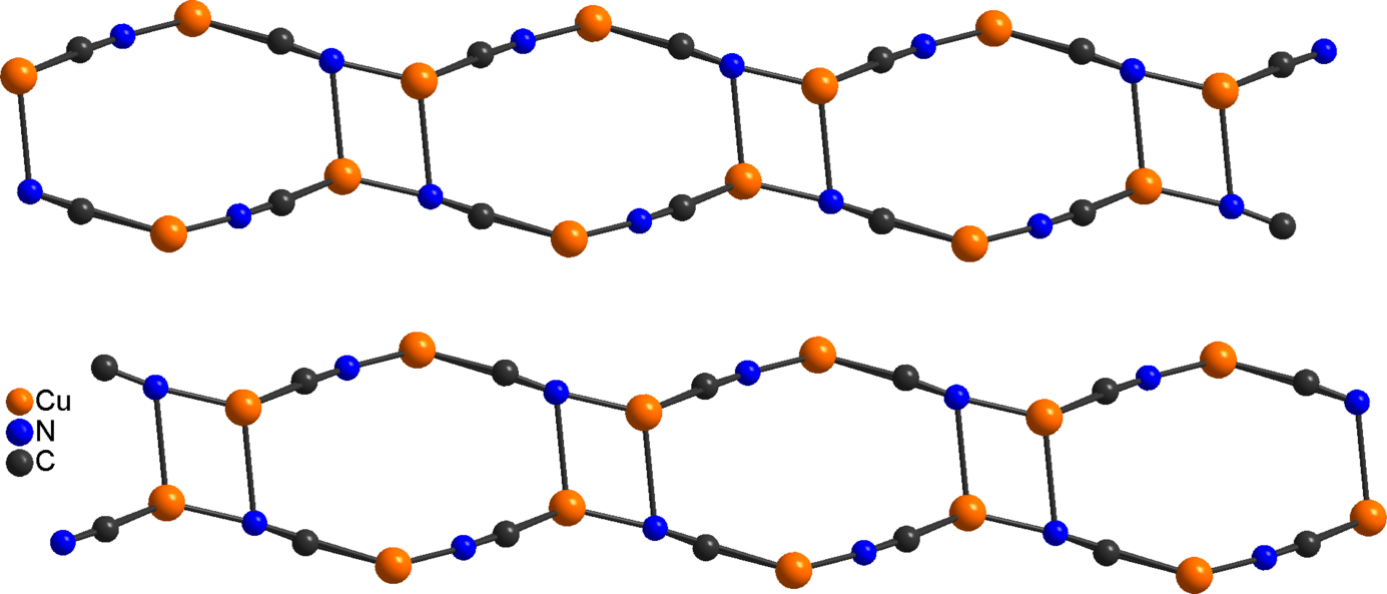
View of the CuCN network in the known isomer of Cu_2_(CN)_2_(2,6-di­methyl­pyrazine) (Chesnut *et al.*, 2001 ▸).

As mentioned in the introduction, some compounds with Cu^I^ halides or pseudo halides and 2,6-di­methyl­pyrazine as ligand are already reported in the CCDC database [Groom *et al.* (2016 ▸); CSD Version 5.43, January 2025; search with CONQUEST (Bruno *et al.*, 2002 ▸)]. These include the second isomer of Cu_2_(CN)_2_(2,6-di­methyl­pyrazine) (Fig. 4 ▸) described in detail in the *Structural commentary* (Refcode SUYGAU; Chesnut *et al.*, 2001 ▸) as well as Cu_2_Cl_2_(2,6-di­methyl­pyrazine (Refcode YEFPOR, Fan *et al.*, 2015 ▸), in which the copper cations are tetra­hedrally coordinated by three μ-1,1 bridging chloride anions and one 2,6-di­methyl­pyrazine ligand. The copper cations are linked by the chloride anions into double chains that are connected into layers by the 2,6-di­methyl­pyrazine ligands. Finally, there is one compound with the composition CuI(2,6-di­methyl­pyrazine) [Refcodes TONQOE (Kitada & Ishida, 2014 ▸) and TONQOE01 (Zhang *et al.*, 2014 ▸)], which shows the same topology of the CuI network as that of the chloride compounds, but in this structure the 2,6-di­methyl­pyrazine ligand is only terminally bonded, which means that a chain structure is formed.

## 5. Synthesis and crystallization

**Synthesis**

Copper(I)cyanide (99%) and 2,6-di­methyl­pyrazine (98%) were purchased from Sigma-Aldrich.

**Figure 5:**
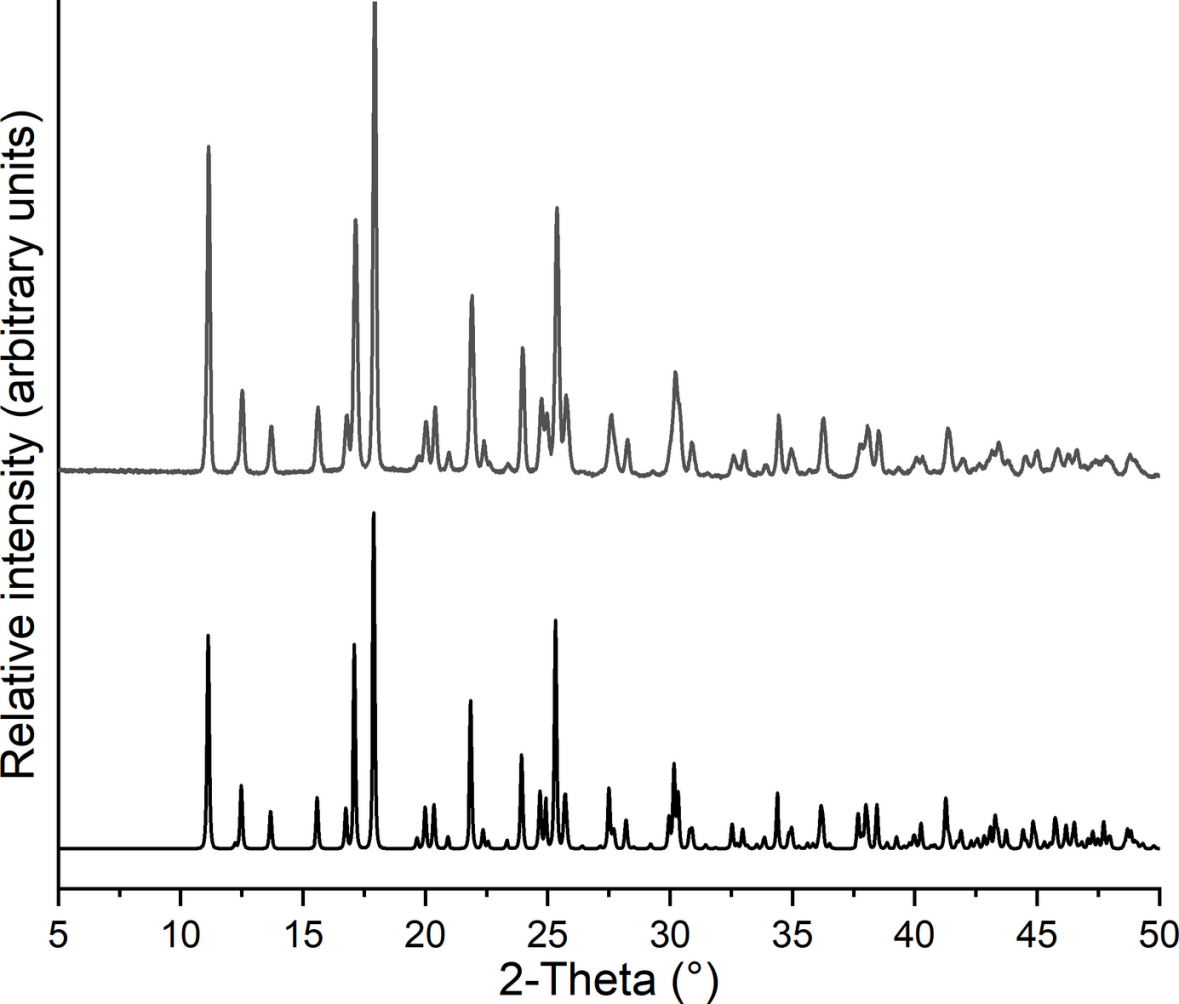
Experimental (top) and calculated X-ray powder pattern (bottom) of the title compound.

179.1 mg (2 mmol) of CuCN and 108.1 mg (1 mmol) of 2,6-di­methyl­pyrazine were stirred in 1 mL of water for 3 d, leading to the formation of a light-yellow-colored microcrystalline precipitate that was filtered off and dried in air. Comparison of the experimental X-ray powder pattern with that calculated from single-crystal data prove that a pure crystalline phase has been obtained (Fig. 5 ▸).

Crystals suitable for single crystal X-ray diffraction were prepared using the same amount of reactants but without stirring.

**Experimental details**

The PXRD measurements were performed with Cu *K*α_1_ radiation (λ = 1.540598 Å) using a Stoe Transmission Powder Diffraction System (STADI P) equipped with a MYTHEN 1K detector and a Johansson-type Ge(111) monochromator.

## 6. Refinement

Crystal data, data collection and structure refinement details are summarized in Table 2 ▸. The C-bound hydrogen atoms were positioned with idealized geometry (methyl H atoms allowed to rotate but not to tip) and were refined isotropically with *U*_iso_(H) = 1.2*U*_eq_(C) (1.5 for methyl H atoms).

The cyanide anions are partly disordered so that the C and N atoms occupy the same crystallographic position. They were therefore refined using EXYZ and EADP leading to a ratio between N3 and C7 and N3′ and C7′ of 94:6 and between N4 and C8 and N4′ and C8′ of 77:23.

## Supplementary Material

Crystal structure: contains datablock(s) I. DOI: 10.1107/S2056989025006267/oo2012sup1.cif

Structure factors: contains datablock(s) I. DOI: 10.1107/S2056989025006267/oo2012Isup2.hkl

CCDC reference: 2472913

Additional supporting information:  crystallographic information; 3D view; checkCIF report

## Figures and Tables

**Table 1:** Selected geometric parameters (Å, °)

Cu1—Cu1^i^	3.0003 (7)	Cu2—Cu2^ii^	2.4031 (7)
Cu1—N1	2.0836 (19)	Cu2—N2^iii^	2.093 (2)
Cu1—N3	1.989 (2)	Cu2—C7^iv^	2.093 (2)
Cu1—C8	1.926 (2)	Cu2—C7^v^	2.125 (2)
Cu1—C8^i^	2.526 (2)	Cu2—N4	1.938 (2)
			
N1—Cu1—C8^i^	112.87 (8)	N2^iii^—Cu2—C7^iv^	107.60 (8)
N3—Cu1—N1	117.84 (8)	N2^iii^—Cu2—C7^v^	105.31 (8)
N3—Cu1—C8^i^	93.53 (8)	C7^iv^—Cu2—C7^v^	110.54 (7)
C8—Cu1—N1	116.40 (9)	N4—Cu2—N2^iii^	104.73 (9)
C8—Cu1—N3	114.87 (9)	N4—Cu2—C7^v^	112.62 (9)
C8—Cu1—C8^i^	96.42 (9)	N4—Cu2—C7^iv^	115.25 (9)

Symmetry codes: (i) 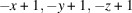; (ii) 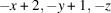; (iii) 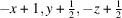; (iv) 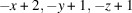; (v) 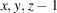.

**Table 2:** Experimental details

Crystal data	
Chemical formula	[Cu_2_(CN)_2_(C_6_H_8_N_2_)]
*M*_r_	287.26
Crystal system, space group	Monoclinic, *P*2_1_/*c*
Temperature (K)	293
*a*, *b*, *c* (Å)	7.0957 (8), 15.8976 (13), 8.1376 (9)
β (°)	92.882 (10)
*V* (Å^3^)	916.80 (16)
*Z*	4
Radiation type	Mo *K*α
μ (mm^−1^)	4.60
Crystal size (mm)	0.2 × 0.05 × 0.05

Data collection	
Diffractometer	Stoe STADI 4
Absorption correction	ψ scan (*REDU4*; Stoe & Cie, 1987 ▸)
*T*_min_, *T*_max_	0.492, 0.558
No. of measured, independent and observed [*I* > 2σ(*I*)] reflections	5136, 2436, 1787
*R*_int_	0.027
(sin θ/λ)_max_ (Å^−1^)	0.682

Refinement	
*R*[*F*^2^ > 2σ(*F*^2^)], *wR*(*F*^2^), *S*	0.026, 0.060, 1.01
No. of reflections	2436
No. of parameters	129
H-atom treatment	H-atom parameters constrained
Δρ_max_, Δρ_min_ (e Å^−3^)	0.49, −0.44

Computer programs: *DIF4* and *REDU4* (Stoe & Cie, 1987 ▸), *SHELXT2014/4* (Sheldrick, 2015*a* ▸), *SHELXL2016/6* (Sheldrick, 2015*b* ▸), *DIAMOND* (Brandenburg, 1999 ▸), *XP* in *SHELXTL-PC* (Sheldrick, 2008 ▸) and *publCIF* (Westrip, 2010 ▸).

## supplementary crystallographic information

### Poly[di-µ3-cyanido-µ-2,6-dimethylpyrazine-dicopper(I)] Crystal data

**Table:**

[Cu_2_(CN)_2_(C_6_H_8_N_2_)]	*F*(000) = 568
*M**_r_* = 287.26	*D*_x_ = 2.081 Mg m^−^^3^
Monoclinic, *P*2_1_/*c*	Mo *K*α radiation, λ = 0.71073 Å
*a* = 7.0957 (8) Å	Cell parameters from 63 reflections
*b* = 15.8976 (13) Å	θ = 12.5–15.5°
*c* = 8.1376 (9) Å	µ = 4.60 mm^−^^1^
β = 92.882 (10)°	*T* = 293 K
*V* = 916.80 (16) Å^3^	Block, ligh yellow
*Z* = 4	0.2 × 0.05 × 0.05 mm

### Poly[di-µ3-cyanido-µ-2,6-dimethylpyrazine-dicopper(I)] Data collection

**Table:**

Stoe STADI 4 diffractometer	*R*_int_ = 0.027
Graphite monochromator	θ_max_ = 29.0°, θ_min_ = 2.8°
ω–θ–scans	*h* = 0→9
Absorption correction: ψ scan (REDU4; Stoe & Cie, 1987)	*k* = −21→21
*T*_min_ = 0.492, *T*_max_ = 0.558	*l* = −11→11
5136 measured reflections	4 standard reflections every 2 h min
2436 independent reflections	intensity decay: none
1787 reflections with *I* > 2σ(*I*)	

### Poly[di-µ3-cyanido-µ-2,6-dimethylpyrazine-dicopper(I)] Refinement

**Table:**

Refinement on *F*^2^	0 restraints
Least-squares matrix: full	Hydrogen site location: inferred from neighbouring sites
*R*[*F*^2^ > 2σ(*F*^2^)] = 0.026	H-atom parameters constrained
*wR*(*F*^2^) = 0.060	*w* = 1/[σ^2^(*F*_o_^2^) + (0.0257*P*)^2^ + 0.2688*P*] where *P* = (*F*_o_^2^ + 2*F*_c_^2^)/3
*S* = 1.01	(Δ/σ)_max_ = 0.001
2436 reflections	Δρ_max_ = 0.49 e Å^−^^3^
129 parameters	Δρ_min_ = −0.43 e Å^−^^3^

### Poly[di-µ3-cyanido-µ-2,6-dimethylpyrazine-dicopper(I)] Special details

**Table:**

Geometry. All esds (except the esd in the dihedral angle between two l.s. planes) are estimated using the full covariance matrix. The cell esds are taken into account individually in the estimation of esds in distances, angles and torsion angles; correlations between esds in cell parameters are only used when they are defined by crystal symmetry. An approximate (isotropic) treatment of cell esds is used for estimating esds involving l.s. planes.

### Poly[di-µ3-cyanido-µ-2,6-dimethylpyrazine-dicopper(I)] Fractional atomic coordinates and isotropic or equivalent isotropic displacement parameters (Å^2^)

**Table:**

	*x*	*y*	*z*	*U*_iso_*/*U*_eq_	Occ. (<1)
Cu1	0.55661 (4)	0.41543 (2)	0.56771 (4)	0.03144 (9)	
Cu2	0.87716 (4)	0.52784 (2)	0.08063 (4)	0.02735 (8)	
N1	0.4099 (3)	0.30232 (12)	0.5414 (2)	0.0225 (4)	
C1	0.5003 (3)	0.22919 (16)	0.5785 (3)	0.0251 (5)	
C2	0.4134 (3)	0.15206 (15)	0.5466 (3)	0.0284 (5)	
H2	0.478870	0.103050	0.574757	0.034*	
N2	0.2396 (3)	0.14575 (13)	0.4772 (2)	0.0255 (4)	
C3	0.1523 (3)	0.21814 (15)	0.4399 (3)	0.0263 (5)	
H3	0.031165	0.216062	0.390699	0.032*	
C4	0.2337 (3)	0.29670 (15)	0.4709 (3)	0.0239 (5)	
C5	0.6974 (4)	0.23279 (18)	0.6514 (4)	0.0372 (6)	
H5A	0.765663	0.276403	0.598490	0.056*	
H5B	0.758464	0.179724	0.635597	0.056*	
H5C	0.694804	0.244561	0.766950	0.056*	
C6	0.1273 (4)	0.37507 (16)	0.4272 (4)	0.0402 (7)	
H6A	0.071258	0.396921	0.523285	0.060*	
H6B	0.030011	0.362659	0.344440	0.060*	
H6C	0.211985	0.416091	0.385614	0.060*	
N3	0.7203 (3)	0.43189 (13)	0.7707 (2)	0.0287 (4)	0.94
C7	0.8343 (4)	0.45479 (15)	0.8634 (3)	0.0281 (5)	0.94
N4	0.7333 (3)	0.48671 (13)	0.2603 (3)	0.0292 (5)	0.77
C8	0.6547 (3)	0.46183 (15)	0.3708 (3)	0.0282 (5)	0.77
N3'	0.8343 (4)	0.45479 (15)	0.8634 (3)	0.0281 (5)	0.06
C7'	0.7203 (3)	0.43189 (13)	0.7707 (2)	0.0287 (4)	0.06
N4'	0.6547 (3)	0.46183 (15)	0.3708 (3)	0.0282 (5)	0.23
C8'	0.7333 (3)	0.48671 (13)	0.2603 (3)	0.0292 (5)	0.23

### Poly[di-µ3-cyanido-µ-2,6-dimethylpyrazine-dicopper(I)] Atomic displacement parameters (Å^2^)

**Table:**

	*U*^11^	*U*^22^	*U*^33^	*U*^12^	*U*^13^	*U*^23^
Cu1	0.02879 (17)	0.03563 (18)	0.02919 (16)	−0.01021 (13)	−0.00544 (12)	0.00005 (14)
Cu2	0.02545 (15)	0.02622 (15)	0.03001 (15)	0.00147 (12)	−0.00232 (11)	0.00123 (13)
N1	0.0193 (9)	0.0250 (10)	0.0228 (9)	−0.0036 (8)	−0.0027 (7)	−0.0003 (8)
C1	0.0194 (10)	0.0259 (12)	0.0295 (12)	−0.0018 (9)	−0.0033 (9)	0.0016 (9)
C2	0.0237 (12)	0.0214 (12)	0.0392 (14)	−0.0001 (9)	−0.0070 (10)	0.0030 (10)
N2	0.0222 (9)	0.0220 (10)	0.0317 (10)	−0.0041 (8)	−0.0046 (8)	−0.0008 (8)
C3	0.0208 (11)	0.0233 (12)	0.0339 (12)	−0.0022 (9)	−0.0070 (9)	−0.0017 (9)
C4	0.0195 (10)	0.0228 (11)	0.0287 (11)	−0.0012 (9)	−0.0047 (9)	0.0011 (9)
C5	0.0263 (13)	0.0330 (14)	0.0507 (16)	−0.0012 (11)	−0.0140 (12)	−0.0005 (13)
C6	0.0336 (13)	0.0203 (12)	0.0644 (19)	0.0008 (10)	−0.0209 (13)	0.0005 (12)
N3	0.0275 (10)	0.0289 (11)	0.0288 (10)	−0.0027 (9)	−0.0058 (8)	−0.0011 (9)
C7	0.0290 (12)	0.0227 (12)	0.0315 (12)	−0.0030 (9)	−0.0093 (10)	0.0046 (9)
N4	0.0275 (11)	0.0264 (11)	0.0335 (11)	−0.0011 (9)	−0.0012 (9)	−0.0001 (9)
C8	0.0260 (11)	0.0288 (12)	0.0293 (11)	−0.0005 (9)	−0.0036 (9)	−0.0029 (10)
N3'	0.0290 (12)	0.0227 (12)	0.0315 (12)	−0.0030 (9)	−0.0093 (10)	0.0046 (9)
C7'	0.0275 (10)	0.0289 (11)	0.0288 (10)	−0.0027 (9)	−0.0058 (8)	−0.0011 (9)
N4'	0.0260 (11)	0.0288 (12)	0.0293 (11)	−0.0005 (9)	−0.0036 (9)	−0.0029 (10)
C8'	0.0275 (11)	0.0264 (11)	0.0335 (11)	−0.0011 (9)	−0.0012 (9)	−0.0001 (9)

### Poly[di-µ3-cyanido-µ-2,6-dimethylpyrazine-dicopper(I)] Geometric parameters (Å, º)

**Table:**

Cu1—Cu1^i^	3.0003 (7)	C2—H2	0.9300
Cu1—N1	2.0836 (19)	C2—N2	1.334 (3)
Cu1—N3	1.989 (2)	N2—C3	1.335 (3)
Cu1—C8	1.926 (2)	C3—H3	0.9300
Cu1—C8^i^	2.526 (2)	C3—C4	1.394 (3)
Cu1—C7'	1.989 (2)	C4—C6	1.491 (3)
Cu1—N4'	1.926 (2)	C5—H5A	0.9600
Cu2—Cu2^ii^	2.4031 (7)	C5—H5B	0.9600
Cu2—N2^iii^	2.093 (2)	C5—H5C	0.9600
Cu2—C7^iv^	2.093 (2)	C6—H6A	0.9600
Cu2—C7^v^	2.125 (2)	C6—H6B	0.9600
Cu2—N4	1.938 (2)	C6—H6C	0.9600
Cu2—C8'	1.938 (2)	N3—C7	1.137 (3)
N1—C1	1.355 (3)	N4—C8	1.152 (3)
N1—C4	1.352 (3)	N3'—C7'	1.137 (3)
C1—C2	1.391 (3)	N4'—C8'	1.152 (3)
C1—C5	1.492 (3)		
			
N1—Cu1—Cu1^i^	127.77 (5)	C2—N2—Cu2^vi^	120.62 (17)
N1—Cu1—C8^i^	112.87 (8)	C2—N2—C3	116.1 (2)
N3—Cu1—Cu1^i^	108.76 (6)	C3—N2—Cu2^vi^	123.17 (15)
N3—Cu1—N1	117.84 (8)	N2—C3—H3	118.4
N3—Cu1—C8^i^	93.53 (8)	N2—C3—C4	123.2 (2)
C8^i^—Cu1—Cu1^i^	39.63 (6)	C4—C3—H3	118.4
C8—Cu1—Cu1^i^	56.79 (7)	N1—C4—C3	120.1 (2)
C8—Cu1—N1	116.40 (9)	N1—C4—C6	119.5 (2)
C8—Cu1—N3	114.87 (9)	C3—C4—C6	120.4 (2)
C8—Cu1—C8^i^	96.42 (9)	C1—C5—H5A	109.5
C7'—Cu1—N1	117.84 (8)	C1—C5—H5B	109.5
C7'—Cu1—C8^i^	93.53 (8)	C1—C5—H5C	109.5
N4'—Cu1—N1	116.40 (9)	H5A—C5—H5B	109.5
N4'—Cu1—C8^i^	96.42 (9)	H5A—C5—H5C	109.5
N4'—Cu1—C7'	114.87 (9)	H5B—C5—H5C	109.5
N2^iii^—Cu2—Cu2^ii^	119.79 (6)	C4—C6—H6A	109.5
N2^iii^—Cu2—C7^iv^	107.60 (8)	C4—C6—H6B	109.5
N2^iii^—Cu2—C7^v^	105.31 (8)	C4—C6—H6C	109.5
C7^iv^—Cu2—Cu2^ii^	55.88 (7)	H6A—C6—H6B	109.5
C7^v^—Cu2—Cu2^ii^	54.66 (7)	H6A—C6—H6C	109.5
C7^iv^—Cu2—C7^v^	110.54 (7)	H6B—C6—H6C	109.5
N4—Cu2—Cu2^ii^	135.36 (7)	C7—N3—Cu1	163.5 (2)
N4—Cu2—N2^iii^	104.73 (9)	Cu2^iv^—C7—Cu2^vii^	69.46 (7)
N4—Cu2—C7^v^	112.62 (9)	N3—C7—Cu2^vii^	142.6 (2)
N4—Cu2—C7^iv^	115.25 (9)	N3—C7—Cu2^iv^	147.3 (2)
C1—N1—Cu1	119.28 (15)	C8—N4—Cu2	177.2 (2)
C4—N1—Cu1	123.18 (16)	Cu1—C8—Cu1^i^	83.58 (9)
C4—N1—C1	117.1 (2)	N4—C8—Cu1	172.1 (2)
N1—C1—C2	120.9 (2)	N4—C8—Cu1^i^	101.84 (19)
N1—C1—C5	118.7 (2)	Cu2^iv^—N3'—Cu2^vii^	69.46 (7)
C2—C1—C5	120.4 (2)	N3'—C7'—Cu1	163.5 (2)
C1—C2—H2	118.8	C8'—N4'—Cu1	172.1 (2)
N2—C2—C1	122.5 (2)	N4'—C8'—Cu2	177.2 (2)
N2—C2—H2	118.8		
			
Cu1—N1—C1—C2	−173.34 (18)	C1—N1—C4—C3	0.2 (3)
Cu1—N1—C1—C5	5.4 (3)	C1—N1—C4—C6	−179.9 (2)
Cu1—N1—C4—C3	172.50 (18)	C1—C2—N2—Cu2^vi^	176.26 (19)
Cu1—N1—C4—C6	−7.6 (3)	C1—C2—N2—C3	−0.2 (4)
Cu1—N3—C7—Cu2^vii^	108.7 (7)	C2—N2—C3—C4	−0.4 (4)
Cu1—N3—C7—Cu2^iv^	−56.4 (10)	N2—C3—C4—N1	0.4 (4)
Cu2^vi^—N2—C3—C4	−176.70 (19)	N2—C3—C4—C6	−179.5 (3)
Cu2^vii^—N3'—C7'—Cu1	108.7 (7)	C4—N1—C1—C2	−0.7 (3)
Cu2^iv^—N3'—C7'—Cu1	−56.4 (10)	C4—N1—C1—C5	178.0 (2)
N1—C1—C2—N2	0.7 (4)	C5—C1—C2—N2	−178.0 (2)

Symmetry codes: (i) −*x*+1, −*y*+1, −*z*+1; (ii) −*x*+2, −*y*+1, −*z*; (iii) −*x*+1, *y*+1/2, −*z*+1/2; (iv) −*x*+2, −*y*+1, −*z*+1; (v) *x*, *y*, *z*−1; (vi) −*x*+1, *y*−1/2, −*z*+1/2; (vii) *x*, *y*, *z*+1.
